# Supplementary material for: Resolving the full spectrum of human genome variation using Linked-Reads
Source: Genome Res. 2019 Apr;29(4):635–45. doi: 10.1101/gr.234443.118 (PMC6442396; doi:10.1101/gr.234443.118)
Supplement: Supplemental Material [file supp_gr.234443.118_Supplemental_longranger-2.2.zip › longranger-2.2.tar.bz2!/lr2.2-public/lib/stan/cmdstan/stan/doc/api/html/categorical_8hpp_source.html]

Stan: src/stan/prob/distributions/multivariate/discrete/categorical.hpp Source File


|  |  |
| --- | --- |
|  | Stan  2.5.0  probability, sampling & optimization |


- Main Page
- Related Pages
- Namespaces
- Classes
- Files

- File List
- File Members

All Classes Namespaces Files Functions Variables Typedefs Enumerations Enumerator Friends Macros Pages

- src
- stan
- prob
- distributions
- multivariate
- discrete

categorical.hpp

Go to the documentation of this file.

1 #ifndef STAN\_\_PROB\_\_DISTRIBUTIONS\_\_MULTIVARIATE\_\_DISCRETE\_\_CATEGORICAL\_HPP

2 #define STAN\_\_PROB\_\_DISTRIBUTIONS\_\_MULTIVARIATE\_\_DISCRETE\_\_CATEGORICAL\_HPP

3

4 #include <boost/random/uniform\_01.hpp>

5 #include <boost/random/variate\_generator.hpp>

6

7 #include <stan/math/error\_handling.hpp>

8 #include <stan/math/matrix\_error\_handling.hpp>

9 #include <stan/math/functions/value\_of.hpp>

10 #include <stan/math/matrix/sum.hpp>

11 #include <stan/math/matrix/meta/index\_type.hpp>

12 #include <stan/prob/constants.hpp>

13 #include <stan/prob/traits.hpp>

14

15 namespace stan {

16

17  namespace prob {

18

19  // Categorical(n|theta) [0 < n <= N; 0 <= theta[n] <= 1; SUM theta = 1]

20  template <bool propto,

21  typename T\_prob>

22  typename boost::math::tools::promote\_args<T\_prob>::type

23  categorical\_log(int n,

24  const Eigen::Matrix<T\_prob,Eigen::Dynamic,1>& theta) {

25  static const char\* function = "stan::prob::categorical\_log(%1%)";

26

27  using stan::math::check\_bounded;

28  using stan::math::check\_simplex;

29  using boost::math::tools::promote\_args;

30  using stan::math::value\_of;

31

32  int lb = 1;

33

34  T\_prob lp = 0.0;

35  check\_bounded(function, n, lb, theta.size(),

36  "Number of categories",

37  &lp);

38

39  if (!stan::is\_constant\_struct<T\_prob>::value) {

40  if (!check\_simplex(function, theta,

41  "Probabilities parameter",

42  &lp))

43  return lp;

44  } else {

45  if (!check\_simplex(function, theta,

46  "Probabilities parameter",

47  &lp))

48  return lp;

49  }

50

51  if (include\_summand<propto,T\_prob>::value)

52  return log(theta(n-1));

53  return 0.0;

54  }

55

56  template <typename T\_prob>

57  inline

58  typename boost::math::tools::promote\_args<T\_prob>::type

59  categorical\_log(const typename

60  math::index\_type<Eigen::Matrix<T\_prob,

61  Eigen::Dynamic,1> >::type n,

62  const Eigen::Matrix<T\_prob,Eigen::Dynamic,1>& theta) {

63

64  return categorical\_log<false>(n,theta);

65  }

66

67

68  // Categorical(n|theta) [0 < n <= N; 0 <= theta[n] <= 1; SUM theta = 1]

69  template <bool propto,

70  typename T\_prob>

71  typename boost::math::tools::promote\_args<T\_prob>::type

72  categorical\_log(const std::vector<int>& ns,

73  const Eigen::Matrix<T\_prob,Eigen::Dynamic,1>& theta) {

74  static const char\* function = "stan::prob::categorical\_log(%1%)";

75

76  using boost::math::tools::promote\_args;

77  using stan::math::check\_bounded;

78  using stan::math::check\_simplex;

79  using stan::math::sum;

80  using stan::math::value\_of;

81

82  int lb = 1;

83

84  T\_prob lp = 0.0;

85  for (size\_t i = 0; i < ns.size(); ++i)

86  check\_bounded(function, ns[i], lb, theta.size(),

87  "element of outcome array",

88  &lp);

89

90  if (!stan::is\_constant\_struct<T\_prob>::value) {

91  if (!check\_simplex(function, theta,

92  "Probabilities parameter",

93  &lp))

94  return lp;

95  } else {

96  if (!check\_simplex(function, theta,

97  "Probabilities parameter",

98  &lp))

99  return lp;

100  }

101

102  if (!include\_summand<propto,T\_prob>::value)

103  return 0.0;

104

105  if (ns.size() == 0)

106  return 0.0;

107

108  Eigen::Matrix<T\_prob,Eigen::Dynamic,1> log\_theta(theta.size());

109  for (int i = 0; i < theta.size(); ++i)

110  log\_theta(i) = log(theta(i));

111

112  Eigen::Matrix<typename boost::math::tools::promote\_args<T\_prob>::type,

113  Eigen::Dynamic,1> log\_theta\_ns(ns.size());

114  for (size\_t i = 0; i < ns.size(); ++i)

115  log\_theta\_ns(i) = log\_theta(ns[i] - 1);

116

117  return sum(log\_theta\_ns);

118  }

119

120

121  template <typename T\_prob>

122  inline

123  typename boost::math::tools::promote\_args<T\_prob>::type

124  categorical\_log(const std::vector<int>& ns,

125  const Eigen::Matrix<T\_prob,Eigen::Dynamic,1>& theta) {

126  return categorical\_log<false>(ns,theta);

127  }

128

129  template <class RNG>

130  inline int

131  categorical\_rng(const Eigen::Matrix<double,Eigen::Dynamic,1>& theta,

132  RNG& rng) {

133  using boost::variate\_generator;

134  using boost::uniform\_01;

135  using stan::math::check\_simplex;

136

137  static const char\* function = "stan::prob::categorical\_rng(%1%)";

138

139  check\_simplex(function, theta,

140  "Probabilities parameter", (double\*)0);

141

142  variate\_generator<RNG&, uniform\_01<> >

143  uniform01\_rng(rng, uniform\_01<>());

144

145  Eigen::VectorXd index(theta.rows());

146  index.setZero();

147

148  for(int i = 0; i < theta.rows(); i++) {

149  for(int j = i; j < theta.rows(); j++)

150  index(j) += theta(i,0);

151  }

152

153  double c = uniform01\_rng();

154  int b = 0;

155  while(c > index(b,0))

156  b++;

157  return b + 1;

158  }

159  }

160 }

161 #endif

stan::math::check\_bounded

bool check\_bounded(const char \*function, const T\_y &y, const T\_low &low, const T\_high &high, const char \*name, T\_result \*result)

**Definition:** check\_bounded.hpp:79

stan::prob::categorical\_log

boost::math::tools::promote\_args< T\_prob >::type categorical\_log(int n, const Eigen::Matrix< T\_prob, Eigen::Dynamic, 1 > &theta)

**Definition:** categorical.hpp:23

traits.hpp

error\_handling.hpp

stan::math::check\_simplex

bool check\_simplex(const char \*function, const Eigen::Matrix< T\_prob, Eigen::Dynamic, 1 > &theta, const char \*name, T\_result \*result)

Return true if the specified vector is simplex.

**Definition:** check\_simplex.hpp:32

matrix\_error\_handling.hpp

constants.hpp

stan::prob::categorical\_rng

int categorical\_rng(const Eigen::Matrix< double, Eigen::Dynamic, 1 > &theta, RNG &rng)

**Definition:** categorical.hpp:131

stan::agrad::sum

fvar< T > sum(const Eigen::Matrix< fvar< T >, R, C > &m)

**Definition:** sum.hpp:14

stan::is\_constant\_struct

Metaprogram to determine if a type has a base scalar type that can be assigned to type double...

**Definition:** traits.hpp:57

stan::math::value\_of

double value\_of(const T x)

Return the value of the specified scalar argument converted to a double value.

**Definition:** value\_of.hpp:24

stan::math::index\_type

Primary template class for the metaprogram to compute the index type of a container.

**Definition:** index\_type.hpp:21

stan::prob::include\_summand

Template metaprogram to calculate whether a summand needs to be included in a proportional (log) prob...

**Definition:** traits.hpp:35

index\_type.hpp

stan::math::sum

double sum(std::vector< double > &x)

**Definition:** sum.hpp:10

value\_of.hpp

sum.hpp

stan::agrad::log

fvar< T > log(const fvar< T > &x)

**Definition:** log.hpp:15


---

[ Stan Home Page ]

*© 2011–2014,
Stan Development Team.*
